# Youth Oriented Activity Trackers: Comprehensive Laboratory- and Field-Based Validation

**DOI:** 10.2196/jmir.6360

**Published:** 2017-07-19

**Authors:** John R Sirard, Brittany Masteller, Patty S Freedson, Albert Mendoza, Amanda Hickey

**Affiliations:** ^1^ Department of Kinesiology University of Massachusetts Amherst Amherst, MA United States; ^2^ Department of Health Science Keene State University Keene, NH United States

**Keywords:** child, movement, fitness trackers

## Abstract

**Background:**

Commercial activity trackers are growing in popularity among adults and some are beginning to be marketed to children. There is, however, a paucity of independent research examining the validity of these devices to detect physical activity of different intensity levels.

**Objectives:**

The purpose of this study was to determine the validity of the output from 3 commercial youth-oriented activity trackers in 3 phases: (1) orbital shaker, (2) structured indoor activities, and (3) 4 days of free-living activity.

**Methods:**

Four units of each activity tracker (Movband [MB], Sqord [SQ], and Zamzee [ZZ]) were tested in an orbital shaker for 5-minutes at three frequencies (1.3, 1.9, and 2.5 Hz). Participants for Phase 2 (N=14) and Phase 3 (N=16) were 6-12 year old children (50% male). For Phase 2, participants completed 9 structured activities while wearing each tracker, the ActiGraph GT3X+ (AG) research accelerometer, and a portable indirect calorimetry system to assess energy expenditure (EE). For Phase 3, participants wore all 4 devices for 4 consecutive days. Correlation coefficients, linear models, and non-parametric statistics evaluated the criterion and construct validity of the activity tracker output.

**Results:**

Output from all devices was significantly associated with oscillation frequency (*r*=.92-.99). During Phase 2, MB and ZZ only differentiated sedentary from light intensity (*P*<.01), whereas the SQ significantly differentiated among all intensity categories (all comparisons *P*<.01), similar to AG and EE. During Phase 3, AG counts were significantly associated with activity tracker output (*r*=.76, .86, and .59 for the MB, SQ, and ZZ, respectively).

**Conclusions:**

Across study phases, the SQ demonstrated stronger validity than the MB and ZZ. The validity of youth-oriented activity trackers may directly impact their effectiveness as behavior modification tools, demonstrating a need for more research on such devices.

## Introduction

The 2008 Physical Activity Guidelines for Americans recommend that children and adolescents engage in at least 60 minutes of physical activity (PA) daily [1]. In the United States, the prevalence of 6-11 year old children meeting this guideline was 42% and drops to 8% for adolescents [2]. This level of PA for the nation’s youth was reported as a “D-” in the recent release of “The 2014 United States Report Card on Physical Activity in Children & Youth” [3]. A low level of PA is one of the behaviors contributing to the current US epidemic of pediatric obesity and to high levels of risk factors for a number of chronic diseases [1].

Exploring novel resources and tools for promoting youth PA are needed. In adults, pedometers and other commercially available PA tracking devices (eg, FitBit, JawBone UP, Misfit Shine) have gained considerable popularity. Similar activity trackers are now being marketed for children. Several studies have now evaluated validity and reliability of adult activity trackers [4-10], but there is currently no independent research validating the output from youth-oriented activity trackers.

Activity trackers are promoted as behavior change tools to increase PA, similar to the way pedometers are used in walking-based intervention studies or community-based programs [11]. Just as pedometers have been validated to support their use as behavior change tools [12,13], these newer activity monitoring devices should also be subjected to validation testing. Use of these commercial activity trackers as behavioral monitoring tools in research interventions is increasing [14,15], with some studies also using these trackers as the assessment tool to determine effectiveness of an intervention [15]. A great degree of caution is warranted in such an approach. Whether used as an intervention or assessment tool, these commercial devices should be sensitive enough to detect different levels of activity (eg, differentiating walking from jogging) in order to provide the user with appropriate feedback. Performing a bout of activity without the activity tracker registering that movement (eg, jogging but only getting “credit” for walking) would make the device irrelevant or even demotivating.

Before being used for intervention purposes, the validity of these commercially available activity trackers needs to be established so that researchers using these devices can be confident in their utility as behavioral tracking devices. In addition, evaluating these youth-oriented activity trackers may help consumers make educated decisions regarding device selection. Therefore, the purpose of this study was to determine the ability of youth-oriented activity trackers to detect the volume of movement in 3 phases, from highly structured to unstructured: (1) using an orbital oscillator, (2) during structured indoor activities, and (3) during 4 days of free-living activity.

## Methods

### Devices

The ActiGraph GT3X+ (AG; ActiGraph, LLC, Pensacola, FL) accelerometer provides an objective estimate of human PA and is used in many research and clinical applications [16-18]. The AG includes a micro-electro-mechanical system (MEMS) based accelerometer with a dynamic range of ± 6 G-forces. The acceleration data are sampled by a 12-bit analog to digital converter at rates ranging from 30 Hz to 100 Hz and stored in a raw, non-filtered accumulated format (G-forces). These data are stored directly into non-volatile flash memory. Raw data are collected at the selected sampling rate and are post-processed in the ActiLife software. Users can generate files containing any desired combination of parametric data (eg, 1 s epoch, 60 s epoch) during the data processing step [19].

**Figure 1:**
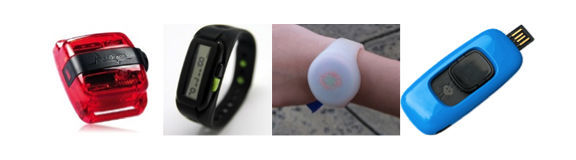
Devices used (from left to right): ActiGraph GT3X+™, MovBand Model 2™, Sqord™, and the Zamzee™.

This study evaluated the output from 3 commercially available activity trackers (see Table 1 and Figure 1). The selection of trackers was based on an Internet search for youth activity trackers. During the time this study was initiated, these were the only devices that seemed suitable for the age range we were targeting. The Movband (MB: Movband, LLC, Brecksville, OH) is a wrist-worn activity tracker that looks like a watch and is similar to many adult-oriented activity trackers. The MB is marketed toward school classroom and Physical Education instructors and also to adult consumers. The face of the unit displays the time and “Moves” or “Steps.” The MB is synced to the associated website by connecting the display piece to the user’s computer via a USB cable. The Sqord (SQ: Sqord, Inc, Durham, NC) is another wrist-worn activity tracker similar to a watch but lacks a display. The Sqord wrist unit syncs the user’s activity “Points” with a computer by tapping the device to a Sync Station that is connected to the computer using a USB cable. Lastly, the Zamzee (ZZ: HopeLab non-profit organization, Redwood City, CA) is a hip-worn activity tracker that uses a built in clip to attach to a user’s waistband, similar to a pedometer. Like the SQ, there is no display. The user syncs the ZZ activity “Pointz” with a computer via a USB cable. All output from the activity trackers (Moves and Steps, Points, and Pointz) are the result of proprietary algorithms; raw acceleration data is not available.

**Table 1:** Descriptions and features of the ActiGraph accelerometer, and the Movband, Sqord, and Zamzee activity trackers.

Device	Placement	Output	Features
ActiGraph GT3X+ (ActiGraph, LLC, Pensacola, FL)	Above right hip; elastic belt	Vector Magnitude Counts from raw (30-Hz) tri-axial acceleration signal	No display; no user interface
Movband Model 2 (Movable, Inc Brecksville, OH)	Dominant wrist	“Moves” based on tri-axial accelerometer and proprietary algorithm	Display screen; upload to website for tracking and group participation
Sqord (Sqord, Inc Durham, NC)	Dominant wrist	Activity “Points” based on tri-axial accelerometer and proprietary algorithm	No display; upload to website (tracking, groups/social network, avatar, challenges)
Zamzee (Hope Lab, NPO, Redwood City, CA)	Above right hip; elastic belt	Activity “Pointz” based on tri-axial accelerometer and proprietary algorithm	No display; upload to website (tracking, groups/social network, avatar, challenges)

### Phase 1: Orbital Shaker Validation

In Phase 1, criterion and construct validity were assessed by comparing activity tracker output to the oscillation frequency and to the AG output respectively. Using the orbital shaker produces a known and constant oscillation frequency, which should be detected by all activity trackers due to the uniformity of the movement. If the devices are not able to detect changes in such structured movement, it would be ill-advised to assume they would work in free-living settings. Therefore, this validation step is included as a first “hurdle” for such devices to clear. Additionally, the highly controlled oscillations allowed for the assessment of inter-unit reliability for all devices.

#### Procedures

An orbital shaker was used to perform electronic motion testing, which produces controlled oscillations between 0.25 and 5.00 Hz. Four trays were mounted on the base of the oscillating plate of the shaker. Each tray had 4 slots to securely position the activity trackers (one of each brand) and the AG (ie, 4 of each device were tested at the same time). All devices were spun continuously for five minutes at three frequencies (1.3, 1.9, and 2.5 Hz) that were previously used to approximate light, moderate, and vigorous intensity physical activity [20,21], and output from each device was summed over the five minutes.

#### Data Processing

Following each 5-minute oscillation frequency, total output for the MB was retrieved from the real-time display and output for both the SQ and ZZ were downloaded and retrieved from their websites. ActiGraph data were collected at 80 Hz, without the low frequency extension, post processed using ActiLife software (version 6.1) and aggregated into counts per second. Since the activity trackers were only able to provide output for the full duration of each activity, an analogous variable was obtained from the AG. Vector magnitude counts from the AG (counts per second output from all 3 axes) were summed over 5 min for each oscillation frequency.

#### Analysis

Spearman correlation coefficients (ρ) were used to assess associations between oscillation frequency and device output (criterion validity) and Wilcoxon Rank Sum tests were used to determine whether there were significant differences in mean output from each device among the 3 oscillation frequencies. In addition, inter-unit reliability was assessed with linear-mixed models to calculate the coefficient of variation in output at each oscillation frequency.

### Phase 2: Structured Activities

In Phase 2, criterion and construct validity were assessed during structured indoor activities by comparing activity tracker output to energy expenditure using a portable indirect calorimetry system and to the AG output.

#### Participants

Children (6 to 12 years old) from the local community were recruited to participate in the validation of the activity trackers during structured activities (see Table 2). Children were included if they had no physical or mental disabilities that would interfere with the child’s ability to perform physical activity or follow protocol instructions. Prior to any data collection, university Institutional Review Board approval and written parent/guardian informed consent were obtained. Children were read an assent script and then, to ensure comprehension, were asked to describe what they would be asked to do for the study.

**Table 2:** Descriptive information for participants in phase 2 and 3.

Participant characteristics	Phase 2: structured activities (n=14)	Phase 3: free living activity (n=16)
Age, mean (SD)	9.0 (2.0)	8.6 (1.6)
Gender (female), n (%)	7 (50)	8 (50)
Height (cm), mean (SD)	135.3 (13.57)	133.9 (12.2)
Weight (kg), mean (SD)	36.1 (10.6)	32.2 (8.4)

#### Devices

In addition to the 3 activity trackers (MB, SQ, ZZ) and the AG, energy expenditure (oxygen consumption [VO_2_] expressed as ml/kg/min) was measured with the Oxycon Mobile portable indirect calorimetry system (Carefusion, Inc.); serving as a criterion measure during the structured activities. The Oxycon Mobile provides breath-by-breath analysis of gas exchange and has been validated for use in children using pediatric-sized face masks and harness [22,23].

#### Procedures

The participants reported to the laboratory on two occasions. The child’s height and weight were measured by trained research staff to the nearest 0.1 cm and 0.1 kg, respectively, using a portable stadiometer (Weigh and Measure, LLC, Olney MD) and digital scale (Seca 876, Hanover, MD). We also obtained age (from date of birth), sex, and grade level at school. Demographic data (age, sex, height, and weight) were entered into each device website as a new user account for each participant. Once in the gymnasium, participants were fitted with the Oxycon Mobile system and one set of activity trackers. The AG and ZZ were placed above the right hip bone on an adjustable belt and the MB and SQ were placed on the dominant wrist as per manufacturer recommendations. The hip-worn AG is the standard placement and has been validated previously using similar activities in similar age groups [24-26] providing the greatest ability for comparison to previous studies.

Ten activities were performed (5 activities per visit). Each activity was performed for 7 minutes, with the exception of quiet sitting, which was performed for 5 minutes. Order effects on EE levels were minimized by allowing for a 5-10 minute break between each activity and by balancing the order of presentation for moderate and vigorous intensity activities. Participants were allowed to remove the Oxycon Mobile mask during breaks and water was provided. A description of the tasks for each visit is provided in Table 3.

**Table 3:** Description of structured activity visits.

Visit 1	Visit 2
1. Sit^a^: quiet sitting	1. Sit: quiet sitting
2. Catch^b^: Stood and played catch, minimal movement (research staff retrieved ball)	2. Cards: sit at table and play cards
3. Self-Paced Walk Instructed to walk at a comfortable pace; Reminded child that there would be a slower and faster walk; Always preceded the Slow- and Moderate-paced walks; Research staff walked with and paced the participant to obtain a constant intensity of effort.	3. Slow Walk: 0.5 miles/hr (0.22 meters/sec) slower than Self-Paced Walk
4. Moderate and Vigorous Activities (Balanced Order) Moderate Walk: 0.5 miles/hr (0.22 meters/sec) faster than Self-Paced Walk Self-Paced Jog: Jog at comfortable pace participant could maintain for 7 min; Research staff jogged with and paced the participant to obtain a constant intensity.	4. Moderate and Vigorous Activities (Balanced Order) Modified Tag: A tag game that involved one researcher playing tag with the participant with a goal to take ribbons off of the belt of the participant-researcher. Modified Relay: 20 meter distance marked on the floor with cones; Researcher demonstrated various calisthenics and movement patterns that involved moving from one cone to the other and back; Participant copied movements; Researcher and participant took turns for duration of activity.

^a^Sit lasted 5 min; all other activities lasted 7 min.

^b^Catch was subsequently classified as moderate intensity due to children frequently not catching the ball and having to walk/jog to retrieve it.

#### Data Processing

After each activity, the data from the activity trackers were uploaded to the device website. Total “Moves,” Points, and “Pointz” for the MB, SQ, and ZZ, respectively, were recorded for each activity. The AG accelerometer data were downloaded via the Actilife computer software (version 6.1). From the raw AG data, total vector magnitude counts for each activity were recorded (analogous to the output from the activity trackers). The Oxycon Mobile data were uploaded and summarized using the system’s software. The computers used to collect the AG and Oxycon Mobile data were synchronized and the first and last minute of the AG and EE data were removed and the remaining five minutes of data was summed; all five minutes of data from the Sit condition were retained.

#### Analysis

Most of the data distributions from the activity trackers were not normally distributed. Therefore, Spearman rho coefficients were calculated to compare activity tracker output to the EE and the AG vector magnitude counts. Since the output variables from each device are different, comparing the absolute output values was not meaningful. Hence, within-device analyses were conducted to examine if the pattern of output from each activity tracker was similar to the pattern observed for EE and AG vector magnitude counts. To limit the number of analyses performed, activities were categorized as Sedentary (both sitting sessions, playing cards), Light (slow walk, self-paced walk), Moderate (catch, moderate walk) and Vigorous (self-paced jog, tag, relay). A repeated measures Wilcoxon Rank Sum non-parametric test was calculated to determine within device differences in output among the intensity categories.

### Phase 3: Free Living Activity

In Phase 3, construct validity was assessed by comparing activity tracker output to the AG vector magnitude counts across all days and for each day.

#### Participants

The same recruitment strategy, inclusion criteria, and informed consent procedures used for Phase 2 were also used for this free living assessment.

#### Procedures

The same demographic and anthropometric data that were collected for Phase 2 (Structured Activities) were obtained from the Phase 3 participants and used for setting up each device. The device placements were also the same as for Phase 2. Children were instructed to wear all four devices during all waking hours for the next 4 full days, except when the devices would get completely wet (eg, showering, bathing, swimming). Participants received verbal instructions and were sent home with a sheet of instructions for all devices and contact information for study staff in the event of technical problems. The AG was initialized to begin recording data at 4:00 AM in the morning after the children received their devices. After the 4 days of wearing the device, children returned all devices.

#### Data Processing

Using the ActiLife software [27], non-wear time was defined as at least 30 minutes of consecutive zeros from the AG data. Data points for non-wear times were set to missing and were not included in any further data processing. Based on the remaining data points, days with less than eight hours of wear time were removed (excessive non-wear time). If a day did not meet this criterion for the AG, all data from the activity trackers were also set to missing for those days. Data points from days with at least eight hours of data were included in further processing. To obtain AG variables comparable to the output from the consumer devices, we calculated the total counts per day using all 3 AG axes (vector magnitude counts). In addition, we also processed the vertical axis AG data using cutpoints developed by Evenson et al [24] to identify total time spent in moderate + vigorous PA (MVPA), and total PA (light + moderate + vigorous). The consumer activity trackers did not allow for extraction of data from specific time points. For the MB, we extracted total “Moves” and steps per day. For the SQ and the ZZ, both provided output as Points or “Pointz” per day, respectively.

#### Analysis

One participant’s data were removed from the data set due to excessive non-wear time on two days and very low activity levels (more than 2 standard deviations below group average) which caused significant skewness in the data. One other participant also had high non-wear time on two days and one other child’s data were missing for one day. Both of these participants were retained for the analyses (removing them did not significantly affect the results). The retained data approximated a normal distribution across all output variables. Since each device uses a different algorithm to process its output, the metrics for each device were different and direct comparisons of absolute values (eg, Moves vs Points/Pointz vs Minutes of MVPA) were not possible. Pearson correlation coefficients were calculated to compare total daily metrics from each activity tracker to analogous metrics from the AG (ie, total vector magnitude counts per day; minutes of total activity and MVPA per day). Repeated measures linear models were calculated for each device separately to identify daily differences in device output.

## Results

### Phase 1: Orbital Shaker Testing

**Figure 2:**
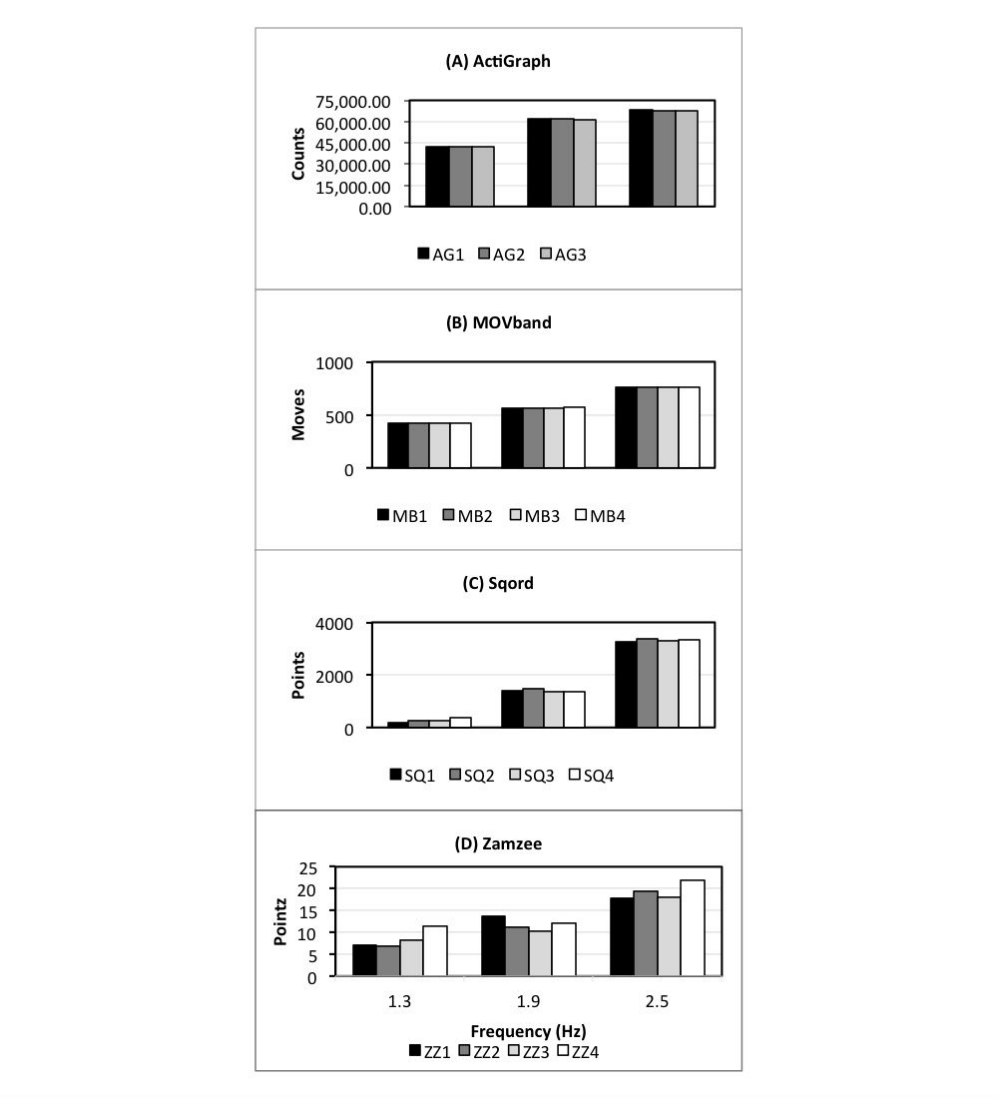
Output as a function of oscillation frequency for individual units of the (A) ActiGraph, (B) Movband, (C) Sqord and (D) Zamzee.

The output for each device was highly correlated with oscillation frequency; correlations for the AG, MB, SQ, and ZZ were .96, .99, .98 and .92, respectively. Output from each device was significantly different among the oscillation frequencies (range *P*=.007-.03). Linear-mixed models revealed that there were no significant inter-unit differences in device output at each frequency (Figure 2). The coefficient of variation at each oscillation frequency revealed that the SQ had the highest variability (29.8%) at the lowest frequency of 1.3 Hz (Table 4). The ZZ displayed the highest variability for oscillation frequencies of 1.9 Hz (12.1%) and 2.5 Hz (9.75%). In contrast, the AG and MB demonstrated lower variability at all oscillation frequencies compared to the SQ and ZZ.

**Table 4:** Coefficient of variation at each oscillation frequency across all units for each device.

Device	Frequency (Hz)		
	1.3	1.9	2.5
AG^a^	0.28	0.41	0.43
MB^b^	0.62	0.85	0.19
SQ^c^	29.8	3.85	1.93
ZZ^d^	25.5	12.1	9.75

^a^AG: ActiGraph.

^b^MB: Movband.

^c^SQ: Sqord.

^d^ZZ: Zamzee.

### Phase 2: Structured Activities

**Figure 3:**
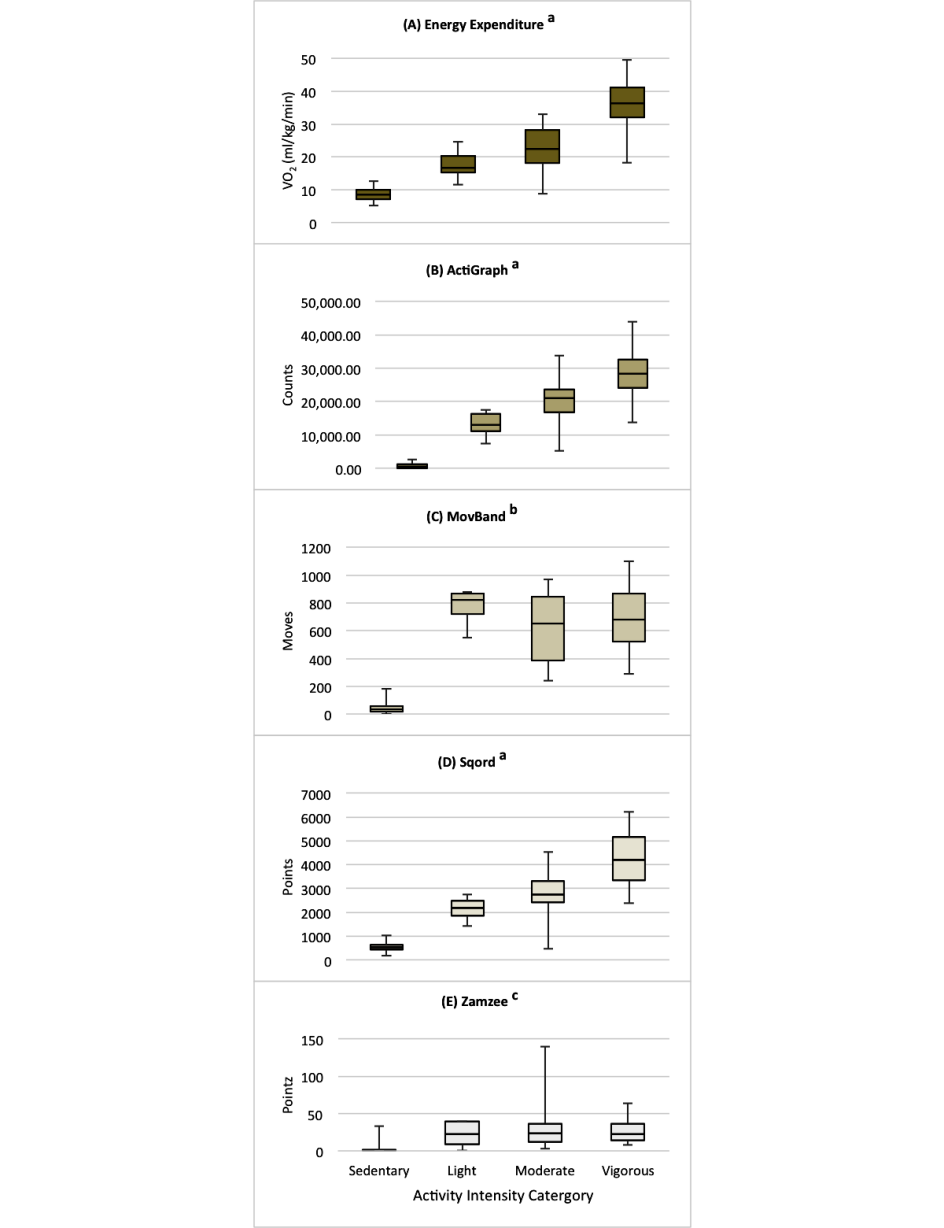
Box and Whisker plots for (A) Energy expenditure, and device output by activity intensity category for the (B) ActiGraph, (C) Movband™, (D) Sqord™, and (E) Zamzee™.

Across all of the structured activities, Spearman correlation coefficients between EE and the AG, MB, SQ, and ZZ were .87, .61, .87, and .60, respectively. Associations among the activity trackers and the AG were .66, .90, and .66 for the MB, SQ, and ZZ, respectively. With activities categorized by intensity (sedentary, light, moderate, and vigorous), EE and AG counts increased in a step-wise fashion (Figure 3 shows Panels A and B, all categories significantly different from each other, *P* ≤.002). Similarly, the SQ demonstrated a step-wise increase in activity points with increasing intensity category (all categories significantly different, *P*<.001, see Figure 3, Panel D). In contrast, the MB differentiated between the sedentary and light intensity categories (*P*<.001, see Figure 3, Panel C) and between light and moderate (*P*=.04), but not between moderate and vigorous (*P*=.32). Of note, the median Moves for the moderate intensity activities were actually lower compared with the light intensity activities. The ZZ only differentiated between sedentary and light intensity activities (*P*<.001) with no difference in ZZ “Pointz” between light versus moderate (*P*=.89), or moderate versus vigorous intensity categories (*P* ≥.67; see Figure 3, Panel E).

**Figure 4:**
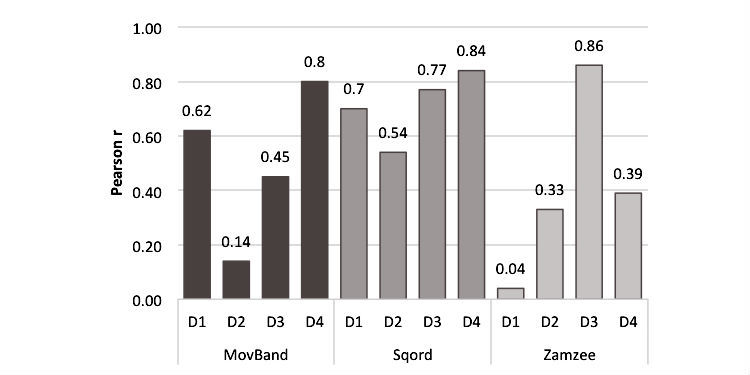
Pearson correlation coefficients with ActiGraph vector magnitude total counts by day for the Movband™, Sqord™, and Zamzee™ activity trackers.

**Figure 5:**
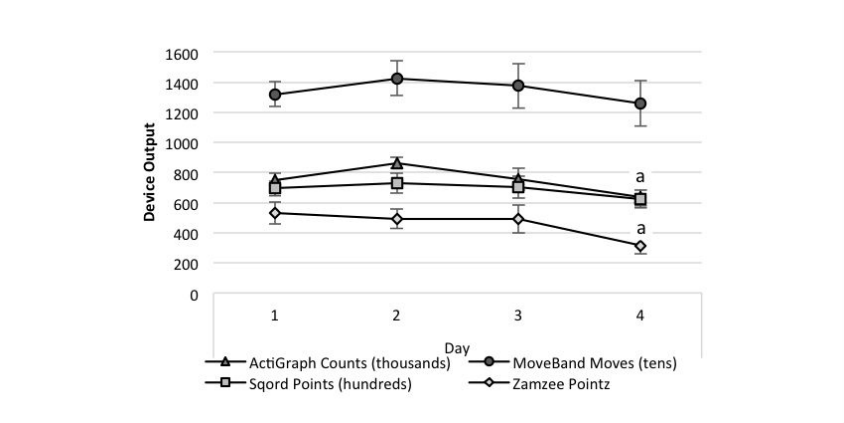
Device output by day. a = Day 4 significantly different from Days 1 and 2 for ActiGraph and Zamzee™ (p <0.04). Note: device output has been scaled to better present all device data in one figure

**Table 5:** ActiGraph minutes of MVPA and total PA per day; mean (SD).

Activity Intensity	Day			
	1	2	3	4^a^
MVPA (moderate +vigorous PA)	57.1 (18.1)	65.5 (15.6)	60.1 (28.7)	48.0 (19.6)
Total PA	157.1 (30.2)	183.1 (28.0)	168.2 (59.0)	144.4 (44.9)

^a^significantly different from Day 2, *P*=.01.

### Phase 3: Free Living Activity

Total vector magnitude counts summed over all days from the AG were significantly associated with the analogous output from the activity trackers (*r*=.76, .86, and .59 for the MB, SQ, and ZZ, respectively). Correlation coefficients between total minutes of MVPA from the AG and the main output variables from the activity trackers were also significant (*r*=.73, .75, .65 for the MB, SQ, and ZZ, respectively). The correlation between output from the MB and SQ, both wrist-worn activity trackers, was high (*r*=.90) while output from the ZZ was more modestly associated with output from the MB (*r*=.71) and SQ (*r*=.73) (all correlations are *P* ≤.005). Day-to-day associations between the AG vector magnitude counts and the output from the MB, SQ, and ZZ ranged from *r*=.14 to .80, *r*=.54 to .84, and *r*=.04 to .86, respectively (see Figure 4).

The total number of steps across all days estimated by the MB was significantly associated with the step estimate from the AG (*r*=.79) and ranged from *r*=.09 for Day 2 and *r*=.68 to .89 across the other 3 days. Compared to the AG, the MB overestimated the total number of steps (AG 34,393 (7128); MB 42,504 (13,764), *P*=.004) and average steps per day (AG 8,865 (1,796); MB 11,055 (2,897), *P*=.03).

The within device repeated measures analyses indicated that for AG and ZZ, the output was significantly greater on days 1 and 2, compared to day 4 (*P* ≤.04). No other day-to-day differences were detected (see Figure 5). Similar results were obtained when performing the same analyses calculated for the minutes per day of MVPA and total PA from the AG. For both variables, day 2 was significantly greater than day 4 (both *P*=.01); no other significant differences were detected (see Table 5).

## Discussion

### Principal Findings

The purpose of this study was to evaluate the validity of 3 youth-oriented commercially available activity trackers using 3 approaches: (1) orbital shaker testing, (2) human testing during structured activities, (3) four days of free-living activity. The major finding of this study was that no consumer device was consistently superior across all 3 approaches to device validation. The SQ, however, performed consistently well during the structured and free-living activities. Compared to the SQ and ZZ, the MB demonstrated the lowest inter-unit variability across all frequencies during the orbital shaker testing; the ZZ demonstrated the greatest inter-unit variability. The ZZ was also less sensitive to the higher intensities performed during the structured activities, and demonstrated lower overall and more variable day-to-day associations with the AG during the free-living phase of this study. However, the ZZ was the only activity tracker that identified lower activity on day 4, compared with days 1 and 2 which was similar to the analysis of the AG counts. With the increased popularity of consumer activity trackers, the use of these devices in research interventions is looming, although based on these findings, caution is warranted. For use by consumers or researchers, activity trackers should be able to differentiate between distinct intensity levels of effort. Performing 30 minutes of vigorous intensity activity but the tracker only registering some or none of the time as vigorous could de-motivate many individuals.

### Comparison With Prior Work

#### Orbital Shaker Testing

Inter-unit variability is an important instrument characteristic when considering the use of these devices in group settings and for tracking intervention progress or change in PA. Since the activity trackers were tightly secured in the orbital shaker, the variability observed is not a function of human variability in movement for a given activity. The greater inter-instrument variability observed for the ZZ will reduce the ability to identify differences between groups or group-level changes over time, requiring larger sample sizes in order for those differences to attain statistical significance. This inter-instrument variability might not be of major concern when examining intra-individual changes. However, these devices are currently being used in group settings (classrooms, physical education) that typically lead to comparisons among children. Thus, two children may perform the same activity but the devices may record different amounts of movement. The relatively high coefficient of variation for the SQ output during the lowest oscillation frequency would indicate that the SQ may not be sensitive to detecting light intensity movements; an important consideration since increasing evidence implicates excess sedentary time as an unhealthy behavior distinct from physical activity [28,29]. During the structured activities, however, the SQ was able to distinguish between the sedentary behaviors (resting, playing cards) and light intensity movement (slow walk).

#### Structured Activities

During the structured activities, the MB and ZZ trackers demonstrated a ceiling effect such that as the intensity of activity increased (according to indirect calorimetry and AG) the output from the trackers plateaued. This could be due to the detectable range for the accelerometer that is used in each device, the sampling and filtering of the raw acceleration signal, and/or the proprietary algorithms used to produce their output (Moves and “Pointz”, respectively). Because these issues are internal to the devices, we are only able to speculate on these issues. Alternatively, the large inter-quartile and overall ranges in the device output for each intensity category indicates substantial inter-individual variability in the output from these devices for the same activities. For example, the self-paced walking and jogging paces were maintained consistently by participants, although each participant’s pace was individualized. Therefore, the inability of the MB and ZZ to distinguish among light, moderate, and vigorous activities may be due, in part, to inter-individual variability, rather than a tracker hardware or software issue. However, the MB and the SQ were both worn on the same wrist with an intensity-related increase observed for the SQ Points but not for the MB Moves. Similarly, while the AG counts increased with activity intensity the ZZ “Pointz” did not increase correspondingly, even though both devices were worn on the hip.

These findings are in contrast to the one other validation study of youth activity trackers. Guthrie et al [30] performed a similar semi-structured protocol with 31, 12-14 year old children (54% female) performing nine activities across a range of intensities while wearing the ZZ and an RT3 research accelerometer. There was a strong association between ZZ and RT3 output (Spearman *r*=.94-.97) and no evidence of systematic bias. The different accelerometer and older age of the children used by Guthrie et al, compared to the current study, may have contributed to some of the between-study differences. One considerable methodological difference between the studies is that Guthrie et al had access to the raw acceleration data from the ZZ allowing them to post-process the raw acceleration data into 10-second epochs for comparison with the RT3 data. This 10-s data is not available with the commercial units used for the current study, thus the reliance on recording total output from the ZZ after each activity. Relying on output metrics based on proprietary algorithms limits our ability to determine if a device is truly valid, since there is no frame of reference for those metrics (eg, Points, Pointz). Having access to the raw data collected by these consumer activity trackers would allow researchers to develop more precise and accurate algorithms, providing information for researchers and users with feedback that would be specific to meeting or not meeting national physical activity recommendations.

#### Free Living Activity

The associations between the activity tracker output and the AG during the four days of free living activity were low and inconsistent for the MB and ZZ, while relatively high and stable for the SQ. One major limitation of all the trackers was that none provided output for minutes per day spent in MVPA, which is the variable that would allow examination of attainment rates for PA guidelines [1,31]. The MB does provide steps per day, and after inputting participant information for device setup, all children were given a 10,000 steps per day goal that could be tracked via the website. The MB also set a 12,000 “Moves” per day goal, although it is not clear if this directly compares to the youth PA recommendation (60 minutes of MVPA per day).

The low and variable day-to-day correlations between the AG and the MB and ZZ trackers may be a reflection of improper device placement and/or inconsistent wear time, although the SQ demonstrated higher and more stable daily associations with the AG. Participants were provided their devices and instructed to wear all 4 consistently at the same time for all 4 days. Access to each activity tracker website was provided as a means of encouraging children to wear the devices each day. However, some participants may have taken off the MB and ZZ to synchronize with the associated website and may not have put it back on in the proper place or may have forgotten to put it back on right away. Additionally, children may not have reattached all of the devices after sleep time. Therefore, the total and pattern of wear time for each tracker and the AG may have been different. Although we provided each participant and his/her parent/guardian with a log sheet to record times when the devices were removed, these were not completed consistently, and some families did not complete it at all. While the ZZ needed to be removed and connected to a USB port to check on progress, neither the MB nor SQ needed to be removed for this task. The periodic removal of the ZZ may have led to children forgetting to put the ZZ back on, leading to wear times that were not consistent with the other devices. Indeed, this participant compliance limitation is present in all free-living studies. Therefore, using a semi-structured protocol would allow an assessment of criterion and/or construct validity by, for example, directly observing children in natural settings (eg, home, playground) while they wear the activity trackers and the AG or other research-grade accelerometer [32].

While the ZZ demonstrated low and variable associations with the AG, it was the only tracker that identified less activity on the fourth day of wear compared to days one and two; similar to the AG. It is likely that the larger variability in the daily values from the MB and SQ limited our ability to detect statistically significant day-to-day differences for these consumer devices. This may be an important consideration if the goal is to use these devices to track changes in activity at the group level, especially for smaller groups of children where inter-individual variability may overwhelm the ability to detect any statistically significant differences between groups or changes over time.

### Strengths and Limitations

To our knowledge, this is only the second study to provide validation results for commercially-available youth-oriented activity trackers [30]. There is a growing body of literature performing similar work with adult activity trackers (eg, FitBit, Misfit, and Jawbone) [4-10]. Given the public health importance of promoting active lifestyles for the nation’s youth, more attention to activity trackers marketed for use by children is needed. To our knowledge, no research has attempted to validate adult-oriented activity trackers worn by children. However, this line of research may be limited since there are additional child protection regulations required for youth activity trackers (eg, the Federal Trade Commission’s Children’s Online Privacy Protection Rule). An additional strength of this study is the comprehensive validation procedures; encompassing orbital shaker testing, and human testing in structured activities and multiple days of free-living activity. We identified potential strengths and weaknesses of each device. Unfortunately, we were not able to accurately assess fidelity with device placement and simultaneous wearing of the devices during the free-living phase of the study. As a result, devices may not have been worn properly, indicating a need for additional research in semi-structured settings. A limitation with all of the trackers is that they employ proprietary algorithms to produce their output and therefore, it is not possible to directly compare output among devices and limits the ability to gauge attainment of PA recommendations.

A limitation endemic to research with activity trackers is the lack of a common metric for direct comparisons among devices. This is due to the proprietary algorithms employed by the device manufacturers. Another possible limitation is the relatively small sample (n=14 and n=16) for the testing of human subjects. Despite this, we were able to observe the expected differences in device output for the ActiGraph, our criterion measure, indicating that we had adequate statistical power to detect meaningful differences. A post-hoc power analysis was performed using G*Power (version 3.1) [33] based on performing Wilcoxon Rank Sum Tests to detect differences between adjacent intensity categories for the structured activities protocol (Phase 2). For the ActiGraph, Sqord, and Zamzee, 80% power was obtained with N=4 to 10 participants, N=4 to 15 participants, and N=11 to 450 participants, respectively. Therefore, the current sample size, while relatively small, was adequate for our purposes.

In summary, all of the activity trackers distinguished between frequencies on the orbital shaker with limited inter-unit variability for the MB and SQ whereas the ZZ units displayed the greatest amount of inter-unit variability. During the structured activities, the SQ was able to distinguish between all of the activity intensity categories, similar to the criterion measures (weight relative energy expenditure and AG vector magnitude counts), while the MB and ZZ did not discriminate among light, moderate and vigorous activity intensity. During the 4 days of free-living activity, the ZZ was the only activity tracker to identify the activity level on the fourth day to be lower than the first and second day, similar to the AG. Visually, the pattern of output from the MB and SQ also resembled that from the AG, but high levels of inter-individual variability prevented the detection of those patterns as statistically significant.

### Conclusions

Of the devices tested, the Sqord demonstrated stronger validity compared with the Movband and Zamzee across study phases. Youth activity tracker manufacturers may use this information to assist with product development and refinement. Interventionists can use this information to assess the utility of these youth-oriented activity trackers as behavior change tools. Physical activity researchers may use this information to conduct additional investigations of other youth- and adult-oriented activity trackers. Future research studies should assess the validity of youth activity trackers in larger, more diverse samples and assess the reliability of these trackers over longer periods of use, which would be important if such devices are to be used as behavioral modification tools.

## Abbreviations

AG: ActiGraph
MB: MovBand
SQ: Sqord
ZZ: Zamzee
EE: energy expenditure
PA: physical activity
MVPA: moderate + vigorous physical activity
